# ‘It is a dilemma’: perspectives of nurse practitioners on health screening of newly arrived migrants

**DOI:** 10.3402/gha.v8.27903

**Published:** 2015-07-22

**Authors:** Faustine K. Nkulu Kalengayi, Anna-Karin Hurtig, Annika Nordstrand, Clas Ahlm, Beth M. Ahlberg

**Affiliations:** 1 Department of Public Health and Clinical Medicine, Division of Epidemiology and Global Health, Umeå University, Umeå, Sweden; 2 Public Health Center, Norbotten County Council, Luleå, Sweden; 3 Department of Clinical Microbiology, Division of Infectious Diseases, Umeå University, Umeå, Sweden; 4 Department of Women's and Child Health, Uppsala University, Uppsala, Sweden; 5 Skaraborg Institute for Research and Development, Skövde, Sweden

**Keywords:** migrants, nurses, language barriers, screening, cultural competence, thematic analysis, health policies, migration policy, interpretive description, Sweden

## Abstract

**Background:**

Screening newly arrived migrants from countries with high burden of communicable diseases of public health significance is part of the Swedish national strategy against the spread of these diseases. However, little is known about its implementation.

**Objective:**

This study aimed at exploring caregivers’ experiences in screening newly arrived migrants to generate knowledge that could inform policy and clinical practice.

**Design:**

Using an interpretive description framework, we conducted semistructured interviews between November and December 2011 in four Swedish counties, with 15 purposively selected nurses with experience in screening migrants. Data were analyzed using thematic analysis.

**Results:**

Participants described a range of challenges including discordant views between migrants and the nurses about medical screening, inconsistencies in rules and practices, and conflicting policies. Participants indicated that sociocultural differences resulted in divergent expectations with migrants viewing the participants as agents of migration authorities. They also expressed concern over being given a new assignment without training and being expected to share responsibilities with staff from other agencies without adequate coordination. Finally, they indicated that existing policies can be confusing and raise ethical issues. All these were compounded by language barriers, making their work environment extremely complex and stressful.

**Conclusions:**

These findings illuminate complex challenges that could limit access to, uptake, and delivery of health screening and undermine public health goals, and highlight the need for a multilevel approach. This entails avoiding the conflation of migration with health issues, harmonizing existing policies to make health care services more accessible and acceptable to migrants, and facilitating health professionals’ work in promoting public health, improving interagency collaboration and the skills of all staff involved in understanding and effectively responding to migrants’ needs, and improving migrants’ health literacy through community outreach interventions.

Migration is a phenomenon characterizing today's globalized world. In 2010, it was estimated that there were around 214 million international migrants worldwide. This figure is expected to exceed 400 million by 2050 (1). The same holds true for Sweden where an increasing trend in the share of foreign-born persons has been observed since the 1940s. The proportion of foreign-born persons was estimated at 15% of the Swedish population in recent official statistics (2). However, this figure does not include vulnerable migrants such as asylum seekers or undocumented migrants. The former does not fulfill Statistics Sweden definition of an immigrant, and the latter is not officially registered. In fact, Statistics Sweden defines an immigrant as a foreign-born person whose actual legal stay will last at least 1 year (3). According to the Swedish Migration Board, more than 81,000 persons sought asylum in Sweden in 2014. The majority originated from countries in conflict with high prevalence of diseases classified as public health threats in Sweden. Most of them originated from the Middle East and North Africa (47%), Sub-Saharan Africa (24%), the Balkans (7%), South and Southeast Asia (6%), former Soviet countries (5%), and South America (0.5%) (4).

The current speed, scope, and complexity of migration make it controversial, and the focus of sensitive debates and growing media attention in many host countries making it a precarious process. Against this background of controversies, many states, including Sweden, are trying to adopt restrictive policies to make migration more complicated, difficult, and unattractive to those in search of security and better lives when they cannot access legal channels of migration. This, however, often results in social and economic environments detrimental to the health and well-being of these vulnerable migrants and the host society (1, 5). For instance, in Sweden, asylum seekers are housed in overcrowded conditions, are spread throughout the country, and their entitlement to care is regulated by a restrictive law (Law 2008:344) (6, 7). With the exception of children (under age 18), asylum seekers and undocumented migrants (since July 2013) have a special entitlement to care that gives them access only to emergency care or care that ‘cannot wait’, the cost of which is covered by the state through the Migration Board (7, 8).

At the same time, there are significant and growing health concerns related to human mobility and the transmission of certain infectious diseases in most receiving countries (5, 9). Evidence suggests that the resurgence and increase in the rates of HIV/AIDS, tuberculosis, and hepatitis have coincided with rising migration flows of people from countries with high prevalence (9, 10). Likewise, in Sweden, the epidemiological profiles of these diseases mirror the global epidemics (11, 12). Nevertheless, disparities in health between native Swedes and those with migrant backgrounds are not limited to communicable diseases. They have been identified in almost all health-related issues (12). Another scare used is the drug resistant strains of various infectious diseases. Consequently, most receiving countries try to contain and eliminate these diseases through legislations and interventions such as medical screening and border control practices targeting migrants from high-burden countries or areas (5, 11). Despite consensus on the utility of screening, its implementation varies in practices among EU/EEA countries (13).

Similarly, the Swedish National Board of Health and Welfare (NBHW) urges county councils or local authorities to offer medical examination or health screening to asylum seekers and other migrants as soon as possible after arrival, in conformity with the Swedish law (Law 2008:344) on health care for asylum seekers and other immigrants (14). Even though a recent law (2013:407) has extended the screening offer to include undocumented migrants, it is not clear whether the concept of other migrants (m.fl.) include students, those coming through family ties, and migrant workers (7, 8). The purpose of the screening is to identify the health and care needs of new migrants, prevent and control communicable diseases of public health significance, and provide information about the Swedish health care system and entitlements to medical and dental care (11, 14). According to the NBHW regulations, information about screening should be provided in a language that the patient understands, and should clearly state the purpose of the screening, that screening is voluntary, and that an interpreter will be used during the screening interview (14). The regulations further stipulate that the health screening shall include an interview to get information about the person's background, possible symptoms, and vaccination history; and to provide information about the Swedish health care. In addition, depending on what emerges during the interview, blood test draws and physical examinations may be performed if necessary (14). However, there are often additional guidelines from county councils on where the screenings should take place, which migrants should be screened, and which tests should be performed resulting in different practices between counties. For instance, some counties offer the screening to all migrants from targeted countries, whereas others limit the offer to some categories of migrants depending on whether the cost will be covered by the state or not (personal communication in a seminar with an infectious disease control officer from the Västerbotten county council, oral communication 17th september 2013). Thus, after receiving information from municipalities or migration authorities about newcomers, the health care staff (often a district nurse) responsible for screening migrants contacts them through a letter notifying them of the offer and the time for screening. The screening is not only voluntary but also free for some categories of migrants as the related costs are subsidized by the state through the Migration Board with compensation to the county performing the screening if carried out within 12 months from the date when the migrant first settled in the county (11). However, available data show that barely half of the asylum seekers undergo the process each year. For example in 2013, only 43% of the 54, 259 asylum seekers underwent the process. This was a decline from the estimated 46% in 2012 (15). Furthermore, little is known about the implementation of this screening program. This study aimed at exploring caregivers’ experiences and perspectives in screening migrants, particularly on issues surrounding the screening of infectious diseases to generate knowledge that could inform policy and clinical practice.

## Methods

### Research design

We adopted an interpretive description (ID) approach to investigate how caregivers engage in screening of migrants in the Swedish context. The idea was to explore questions related to implementation and to generate knowledge that could advance disciplinary knowledge and inform practice, rather than theory development or general qualitative description (16). ID is designed to explore real-world situations in which participants are not able to fit together their lines of action, where definitions of the situation are insufficiently shared, or where common definitions lead to action no longer useful (16). This applies to the current inquiry where people with different backgrounds and therefore perceptions of the situation interact through a complex set of structures and policies (migration and public health laws). The rationale for using ID approach was to explore how the above factors influence the implementation of health screening. We are particularly interested in how caregivers implement and manage the screening program within the Swedish environment. The research questions that guided this study include: target groups for screening, the screening process, challenges in screening migrants, norms and regulations in screening migrants, and common barriers for access and use of screening services.

### Study settings and participants

The first author purposively recruited participants at selected primary health care centers in four counties in Northern Sweden through a professional network *Kunskapsnätverk Hiv/STI Norr* (the Northern HIV/STI's knowledge network). Nurses who commonly screen migrants were then approached by email or telephone calls and, after being informed about the study, were asked if they would like to participate. The recruitment continued until no new ideas were generated. The 15 participants were all female aged between 27 and 64 years, predominantly district nurses (*N*=13), and two public health nurses. The majority were native Swedes (*N*=12). All participants were interviewed in Swedish except one who preferred English.

### Data collection

The first author interviewed the 15 nurses who agreed to participate in the study at their offices between November and December 2011, each interview lasting 60–90 min. The following open ended questions were used with follow up questions:

- What is medical screening and what is it used for?
- Who are eligible for the screening program?
- Could you please, describe the screening process?
- How does it feel to screen people with migrant backgrounds?
- What are the challenges in screening people with migrant background?
- What are your experiences working with interpreters?

Demographic data were also collected and included age, sex, location, and occupation. Each interview was recorded using a Dictaphone, transcribed verbatim, and read through to inform the ongoing data collection process. The main investigator (first author) also wrote supplementary field notes and appended preliminary analytical notes to each interview transcript.

The first author, a medical doctor with migrant background, conducted this inquiry not only in her role as an academic and researcher but also in her role as a migrant, a cultural mediator, and health professional. Thus, she could identify herself as both an insider and an outsider. All these roles undoubtedly affected and shaped her way of being in terms of the assumptions and biases that she brought to the study. However, her position as a researcher that also included reviewing other literature and participating in academic conferences on the research topic helped to shape her perspectives through critical awareness of the self (reflexivity). This method allows researchers to reflect on and to critically question their assumptions to deal with biases.

#### Ethical considerations

Besides the formal ethical approval for the research project from the regional ethical committee at Umeå University, we obtained informed consent prior to each interview. Names mentioned in interviews were removed, as was anything which made the transcript identifiable to a person or a medical setting.

### Data analysis

Using a constant comparative and thematic analysis approach, the first (FKNK) and last authors (BMA), both with migrant backgrounds, read through the interview transcripts and recorded observations separately, to find patterns and commonalities between interviews while maintaining attention to individual variation. They thereafter compared, discussed, and reached agreement on differences in meaning of codes and emerging patterns. Three other Swedish members of the research team and co-authors of this article (AKH, AN, CA) also participated in the discussions and acted as point of reference for the issue under investigation. The meanings of codes were refined through the process and linked to create categories (17). A summary of the preliminary results was presented and discussed with the screening staff (nurse practitioners) in different settings, including the study settings. Finally, by linking the categories/subthemes that emerged at the descriptive level, three themes that make sense of what caregivers said about screening migrants were developed. They include: 1) discordant views about medical screening, 2) inconsistencies in rules and practices, and 3) conflicting policies.

## Results

The following section highlights the views of participants about the challenges they encountered in screening migrants and how these affected the screening process. Each of the three themes will be discussed separately and illustrated with quotations from interviews.

### Discordant views about health screening

#### Differing perceptions and expectations

Participants stressed that some of the challenges in screening lie in the discrepancies between their understanding of the health screening and that of migrants. This was described as stemming from sociocultural differences thus resulting in divergent expectations. They described, for example, being challenged by low educated migrants with poor knowledge of health issues, making it difficult for them to understand what was going on. One nurse stated, *When you meet these low educated ones, those who have not attended school. It is sometimes difficult to lower the level. I think that it is the toughest thing* (N12). Participants stressed that such people hardly believed that they could carry an infection without symptoms. One nurse, apparently shocked by this limited knowledge, commented: *Well, this hepatitis B, it seems to be quite natural to have it. It's nothing dramatic* (N5). Other participants added that some migrants denied latent infection diagnoses because they expected to have symptoms. They thus blamed the screening process ‘for making them sick’. According to participants, this sometimes led to conflicts as these patients sometimes requested immediate treatment after being told that they carried an infection. In addition, other participants were concerned about the impact of cultural beliefs, practices, and past experience on migrants’ attitudes toward health screening and immunization, which in some countries is perceived as dangerous or unnecessary as one nurse commented:

There are people who have refused to take blood tests either on themselves or on their children … do not … want any vaccination assessments for the children. However, they feel confident anyway because they had heard that it was dangerous in their homeland …. (N1)

Other cultural differences believed to affect health screening were sex segregation and the meaning of time. Some participants claimed that migrants had negative attitudes toward providers of the opposite sex. One nurse said, *There was a father who did not want to shake my hand. This was a remarkable experience. I could not help feeling undervalued, though he kindly explained to me that it is to do with his culture* (N14). Another added, *We have a male counselor that women do not always go to because he is a man, and they want female doctor, female interpreter* (N3). Other participants stressed that the difference in meaning of time was a big challenge too, as migrants were often late and seemed not to worry about the consequences. One nurse commented, *I simply believe that it is because we have completely different cultural backgrounds. Our whole society is built around being on time, which might not be the case for theirs* (N6).

#### Health care professionals or immigration authorities?

The participants also expressed the view that migrants sometimes mistakenly viewed them as migration authority figures. They argued that no matter how hard they explained that they were only health caregivers working independently and under the obligation of professional secrecy and confidentiality; they felt that migrants, particularly asylum seekers, were concerned that they collaborated with the Swedish Migration Board to their detriment and thus became uncooperative as indicated by one nurse:

They believe we are government officials who might disclose things they do not want to come out to someone who can expel them. … I have noticed a difference. … Quota refugees are much more outspoken and will tell you more. Many asylum seekers say they do not know. That is their answer to many questions …. (N4)

Other participants who shared this view elaborated on this anxiety and said the caregivers believed screening would facilitate access to appropriate care, but they sensed that migrants believed a positive result could negatively affect their prospect of getting asylum and instead lead to deportation. The participants’ perceptions of health screening as a benefit thus differed from that of migrants who perceived it as a process of scrutiny to obtain permission to stay as indicated below:

People should understand that it is not an inspection/scrutiny in order to get into the country, but it is like an offer from the country so that we can complete the vaccination status and check that they are fine when they arrive and offer the help that may be needed …. (N2)

Another divergent view was that the participants considered migrants to be vulnerable to poor health and expected them to cooperate regarding the health screening offer. Instead, they felt some migrants perceived themselves as healthy and did not understand why they should undergo health screening. The participants argued that such people did not prioritize health in general, particularly health screening, because they had their own priorities or competing needs as indicated by one nurse:

This health screening is a secondary issue. They have so many other concerns and may think: ‘Well, there is nothing wrong with me, I need help with other things and I am therefore not interested in health screening’ …. (N3)

Such patients were described as suspicious and questioned the health screening offer, especially when they realized that some of the questions asked during the screening were similar to those asked by the Migration Board during the asylum interview.

### Inconsistencies in rules and practices

#### New assignment, but lack of training and resources

Participants spoke of inconsistencies in the official discourses and their daily practices. Several reported that they were assigned to a new task, but they lacked appropriate training and skills to perform it. They reported feeling limited in their knowledge, skills, and understanding of culturally diverse patients. They indicated, for example, that it was difficult to talk about sexual health and traumatic experiences with migrants because they did not know what to say and how to say it without offending the patient. They stressed they often had to learn from their mistakes as indicated by one participant:

I was thrown into it directly. I think some training would … be useful. Because I have noticed a big difference … When I do it now against then when I started. I have learnt over time. So I think some training would have been good …. (N5)

The participants strongly expressed concern over being expected to screen newly arrived migrants within 2 months, because there were not enough staff at their units to do the screening within the established time frame. They complained that often there were only one or two nurses for the task regardless of the number to be screened. This, according to them, led to overwhelming time pressure and frustration because they received no help from colleagues for other duties besides screening. One nurse expressed her frustration in the following way:

If one should like run two different services at the same time, it becomes a hassle in the long run, and then the quality of the service will certainly become poor because you do not have time to do what you are supposed to do. It’s frustrating …. (N7)

Participants also reported that the screening units remained closed during vacation because colleagues on duty did not want to take over due to fear or simply negative attitudes and prejudices toward migrants. Talking about her colleagues’ attitudes, a nurse resentfully stated, *Colleagues think it is strange, and there is fear because it is strange, unnecessary fear and prejudice* (N8). However, other participants argued that the problem was that most migration inflows often took place at times when the primary health centers were actually understaffed so that some units, including the health screening units had to be kept closed. One nurse said, *They often arrive at … times when the health center is poorly staffed. … Then there are fewer people working so some actually undergo their health screening beyond the reasonable time* (N2).

#### Many people involved, but poor coordination

Participants reported that they were expected to share responsibilities with staff from other migrant-serving agencies, but there was poor coordination which delayed the process. There was consequently complaint that even the allocated time for screening was not enough because there was a lot to be done and working with an interpreter added to the problem. One participant explained:

Each refugee patient takes much more time than a Swedish patient … partly because there is an interpreter and also because there are many people involved. We're supposed to report and often collaborate with so many other public institutions, which take times …. (N12)

Participants further argued that the involvement of many people and services was not well coordinated. This resulted in breakdown in communication. They indicated that information about arriving migrants and requests for screening emanated from different sources depending on the type of immigration, and that in some cases they received contradictory information and spent a lot of time trying to sort out things. One nurse complained:

So they have a lot of people that interfere … sometimes you get different reports from various individuals about the same patients. … Then you cannot really know what is what until you bring the patient here …. (N11)

Participants further expressed concern over not getting information about all people arriving in Sweden, which made it difficult for them to offer health screening to all migrants from targeted countries.

We never get information about migrant workers and other family ties … It is like nobody is responsible for informing us. Even for those we usually get informed about by the municipality, it happens that they forget to inform us about newcomers …. (N14)

However, it was also argued that migrants sometimes missed their appointment not because they did not want to be screened, but because the invitation sent out did not reach them for various reasons as indicated below:

… Most migrants want to come. My experience is that when they have not come, it is usually because we got wrong information about the address or the mail was not delivered or that the name was not written on the door. It's usually such hurdles. I do not have the feeling that they do not want to come. In fact, it is quite the opposite. It is very seldom that somebody declines the offer …. (N7)

Some participants added that even when the notice reached them, migrants still missed their screening appointments because they often had concurrent appointments at the Migration Board or the lawyer, which they naturally prioritized. While speculating about why some migrants did not show up, a nurse said: *those who have not come, it often turns out that they have been unable to do so. They have been at some other appointments* (N10). Participants expressed concern over long delays, which they believed could undermine the goals of screening. One nurse commented:

What does not feel good right now with this screening is the long waiting time. I do not think that it is okay. People can still carry some diseases that they do not feel sick from, and yet still they may infect others …. (N6)

#### Language barriers and the complexity of working with interpreters

Another discrepancy mentioned was the practice of sending invitations for health screening in Swedish to arriving migrants despite general awareness of their inability to read and understand Swedish. Most participants, however, argued that migrants were already given information about the health screening by the Migration Board staff, as one participant remarked: *Actually, they have been briefed about it at the Migration Board that they will be called for medical examination and that the notice will be sent in Swedish* (N12). Other participants further added that recently arrived migrants could also get help from friends, relatives, or other people from their own communities to read for them. Some participants, however, expressed their frustration over this practice and stressed that they were reluctantly following the Swedish Migration Board request as explained below:

I asked the Migration Board when I started working with this because I thought … I might be able to send the notice in any language. But they told me: ‘we don’t do that and we do not think you should do it either, you should send notices in Swedish because they always have someone they can ask’ …. (N5)

Other participants argued that even though it felt inappropriate, the majority of migrants actually came at their appointed times. One nurse said, *They may not be able to read what is written on the notice. But, my experience is that there are many people who cannot read and write; still they come* (N10).

The participants, however, admitted that language and literacy were important barriers as migrants might not be able to read the notice and understand when and where they should turn for health screening. The participants also stressed that, even though the law gives all new migrants the right to language assistance, working with an interpreter was not only time consuming, but also complicated. The participants complained about the lack of interpreters for certain languages and indicated that in some areas, they had only access to telephone interpreters. According to participants, even when interpreters were available, communication problems persisted due to lack of competent interpreters, patient discomfort for political or psychosocial reasons, limited-time to use the interpreter, and the difficulty in finding an interpreter with the appropriate dialect within a language, sex, or country of origin while booking an interpreter. Some interpreters were described as unprofessional, whereas others were said to have little knowledge of medical terms. When asked about her experience with interpreters, a nurse angrily answered: *A few are actually good. But, some are a total disaster. They interfere in the conversation somehow* (N10).

### Conflicting policies

#### Individual or population needs?

Participants had divergent opinions regarding the purpose of the health screening. Some emphasized the need to protect the host population by identifying infectious diseases of public health significance to prevent them, whereas others emphasized the importance of identifying individual health needs and providing appropriate care. However, the health screening was described as mainly focused on identifying infectious diseases of public health significance so defined in the official guidelines and recommendations developed by county infectious disease control officers who also determined which tests should be carried out. A nurse explained: *We do not carry out various tests to identify all kind of diseases. It is not part of our mission. Instead, it's our county infection control officer who has determined which tests we must carry out* (N6).

Participants stressed that the narrow focus on infectious diseases often led to conflict with migrants because the latter had high expectations and thus required all their health needs to be assessed and met. The participants also argued that this narrow screening policy carried the potential to portray migrants as disease vectors, and indicated that in some areas, children were not allowed to attend school if they were not screened. They indicated it also made some migrants feel offended, discriminated against, and reluctant to discuss sensitive health issues such as HIV or their sexuality. One participant explained:

They feel offended like: ‘you talk about HIV with me just because I am from somewhere else’. That's what I sometimes get as response when I try … I guess it is like: ‘you think that anyone who comes from another country has HIV’ and then they get pissed off, so that it has been a little bit difficult to approach these questions …. (N8)

#### Managing health or controlling immigration?

The participants expressed the view that the restrictive migration law that limits the entitlement of some categories of migrants only to ‘care that cannot be postponed’ was not only a potential source of conflict, but also an ethical dilemma. They claimed that they often ended in conflict with asylum seekers who often questioned this law and required care that they were not entitled to. They even argued that it was not clear to them as to how the law defines ‘care that cannot be postponed’. Talking about the challenges in screening asylum seekers one nurse said: *I cannot help them as much as they wish. They have high expectations but limited access to care and the line is somewhat hazy* (N8).

They further described their frustration for not being able to help or follow up asylum seekers who had mental health problems or other chronic conditions because of the migration law. They expressed facing a dilemma because this law conflicted with their code of ethics. Talking about the law, one participant said: *I think this is a paradox, because health is more than the absence of acute conditions* (N14). Some participants talked about their attempts to circumvent the law and provide treatment. Others questioned the screening of asylum seekers and argued that it raised ethical concerns because they were supposed to tell them the diagnoses of non-acute condition, but then deny them treatment or interrupt some treatment before they were properly treated if they were deported by the Migration Board.

Participants also expressed concern over the Migration Board's housing and dispersal policy for asylum seekers. This policy was said to make it difficult not only for them to reach asylum seekers with information about screening, but also for the migrants to reach the screening unit. They described how asylum seekers were spread out in remote areas, some far from the nearest health care unit. They argued that the Migration Board staff cared more about the availability of accommodation rather than the accessibility of health care. According to them, distance was an obstacle to both screening and care because some migrants were often late for the appointment or simply did not turn up as they could not find their way to the health care center. In this way, they argued the Migration Board made asylum seekers unreachable as they moved them from place to place without notification.

The participants also expressed concern over the housing situation for asylum seekers who lived in overcrowded conditions, which often resulted in constant fear of forced disclosure among those diagnosed with stigmatized conditions such as HIV and TB. Such overcrowding was also viewed as a barrier to the control of communicable diseases.

## Discussion

This study illuminates the complex challenges limiting access, uptake, and delivery of medical screening as well as other curative and preventive services. The challenges described include discordant views and expectations about health screening resulting in misunderstandings during the screening, and preventing the development of a trustful relationship. In addition, inconsistencies in official discourse and practice as well as conflicting policies created a complex and demanding working environment. This was compounded by language barriers and lack of appropriate competence on the part of care providers. All these challenges carry the potential to hamper the implementation of health screening and the achievement of its public health goals. These findings have significant implications.

### Implications for clinical practice

This study indicates that sociocultural differences between providers who participated in this study and migrants often translated into divergent views and expectations that created tensions during the screening encounter. Cultural differences have been mentioned in the literature as a source of misunderstandings and a barrier to access and provision of care to migrant patients (18–20). Apart from cultural diversity and related challenges, low education status among migrants was said to influence knowledge about health issues, leading to limited comprehension, ineffective communication, and poor participation in health screening and vaccination. This study confirms what has been described in other studies emphasizing the need for strategic and evidence-based health education interventions to reach and educate these vulnerable populations (21–23). However, it should not be forgotten that the language of medicine is highly technical and complex, which makes it difficult for outsiders to comprehend medical terms commonly used in the health care environment (21, 24, 25). Nevertheless, speaking different languages also contributes significantly to the challenges faced by participants during the screening encounter. Contrary to widely held beliefs that this issue is readily addressed with a legal right to interpreters, our findings suggest that communication problems persist, reflecting sociocultural differences, wide gaps in the availability of interpreter services, and knowledge gaps on the part of available interpreters. A previous study found that language barriers negatively affected the quality of communication and symptoms reporting during screening (26). Other studies have also identified language barriers as a substantial challenge in providing care to culturally and linguistically diverse patients (19, 20, 22, 27). Moreover, the fact that asylum seekers consider health care professionals as officials of the Migration Board creates suspicion and mistrust that may negatively affect the patient–provider relationship and limit access to and use of the screening service. This was compounded by the striking similarity between the screening and Migration Board interview, competing needs, and lack of perceived benefits.

### Implications for capacity building and resource allocation

This study further suggests that lack of appropriately trained staff inhibited inquiry about delicate questions and discussing sensitive health issues with migrants as reported in another study (28). Lack of cultural sensitivity and underinvestment in culturally competent services have been identified as potential barriers to screening services for culturally diverse patients (18). Despite the claim for a multicultural approach to migration in Sweden, participants in this study described a mismatch between this official discourse and the harsh realities of their everyday practices. The personnel in the health screening service lacked the required training in addition to the shortage of staff and time. They reported not only their own, but also colleagues’ lack of knowledge and training in providing care to culturally diverse patients, and expressed their need for training. The training needs were also reported by nurses who participated in other studies (19, 27). Moreover, evidence shows that many care providers lack appropriate competence and knowledge in caring for culturally diverse patients (19–21). The attitudes and degree of training of health professionals and other staff working with migrants are major determinants of migrants’ likelihood to utilize health services efficiently (10, 29). Because the screening is often the first contact migrants have with the Swedish health care system, it is vital that nurses who screen migrants get appropriate training and develop competence, to avoid high expectations and a poor image that could impede future utilization (23). Nevertheless, training staff may be challenging due to the diversity of migrant groups, the changing patterns of migration over time, and lack of standards. These issues require further investigation. Moreover, the shortage of staff and time, in addition to heavy work load, led to time constraints and closure of screening units during critical periods, which resulted in long waiting times. This raises serious questions about the quality of screening and the extent to which public health goals are being achieved. Other studies have similarly identified inadequate resources and the stressful work environment it creates as major barriers to providing culturally competent care (19, 27).

### Implications for planners of screening programs

This study also illustrates breakdowns in the structure, coordination, and implementation of the health screening service that could hamper the process and undermine its goals. There were breakdowns in communication between the services involved in the screening process, as well as between migrants and care providers. Others breakdowns reported in this study include inaccurate information, not being notified about address changes, competing roles and responsibilities, and overlapping appointments. All these were believed to result in delayed or missed appointments. Moreover, despite awareness about migrants’ limited Swedish proficiency, participants reported that they sent notices about screening in Swedish to newly arrived migrants. Although some participants elucidated that this was requested by the Migration Board, this practice is inconsistent with the NBHW recommendation, which stipulates that notice about screening should be provided in a language the patient understands (14). Participants who endorsed this practice argued that most migrants actually attended the screening appointment. However, it cannot be ascertained whether those who came did so because they could read and make sense of the notice or because they believed the screening was mandatory or necessary to them. Yet, undergoing health screening might be perceived as a way to abide by the rules of the host country on residence, which might explain the high attendance rate reported by some participants despite language and literacy barriers. The question is whether it is appropriate to send written notices in Swedish to people whose literacy and language skills are unknown. Even less effective is reliance on other migrants or Migration Board staff who are not health professionals to translate or provide information about health screening. Moreover, the involvement of migration staff may explain the fear and suspicion about an eventual connection between the health screening interview and that of the Migration Board and foster the confusion about the motivation behind health screening or the role of health care professionals. This connection has also been reported in other studies that described it as detrimental to the development of a trustful patient–provider relationship (27, 28, 30). All these can impede the screening process, inhibit trust and openness during the screening interview, and lead to withholding of important information, misdiagnosis, or delayed diagnosis with negative consequences for individuals and society. Moreover, it has been stressed that the context in which screening is offered and who offers it are important determinants of test acceptance (18).

### Implications for policymakers

This study indicates that existing policies are full of paradoxes and ambiguities, creating ethical dilemmas for health care providers. The first issue is the focus on infectious diseases, which might not accurately reflect the actual needs of migrants or accurately reflect the differences in needs among migrants (5, 22). Participants in this study argued that migrants, particularly asylum seekers and refugees, often experienced traumatic events before and during migration and had as much needs for mental health as for infectious diseases care. But, they were unable to concentrate on the former because the focus of the screening guidelines is identification of infectious diseases of public health significance, which frustrated both migrants and themselves. Although justified, the focus on infectious diseases created tensions and hindered providers from discussing stigmatized conditions such as HIV due to feelings of discrimination. This can result in prejudices and exacerbates discrimination against targeted migrant groups in the wider society, despite limited evidence of transmission between migrants and native born (9, 10). The fact that migrant children were reported not to be allowed to start school until they were screened is an illustrative example of the wider society understanding and interpretation of the goals of the screening (31). This approach has been criticized as it may create a false sense of security among natives that only migrants are at risk of infectious diseases and divert attention from addressing actual determinants of health inequalities between migrants and natives (22, 32). The stigma and discrimination related to infectious diseases can exacerbate the social exclusion of migrants and act as disincentive to undergoing health screening, delay or hamper early diagnosis and treatment, and compromise public health goals. According to a recent report, late HIV diagnosis in migrants is a key issue in many European countries, including Sweden (9). The second issue is targeting of migrant groups. Except for one, the screening program in counties included in this study only focuses on asylum seekers and refugees and seems to overlook other migrant categories from targeted countries who do not qualify for state compensation (33). Moreover, undocumented migrants are not officially registered and may be hard to reach or deterred from responding to the screening offer for fear of being reported to the migration authorities (30). From an infection control standpoint, the screening program may need to encompass all migrants from targeted countries to achieve its goals. This study also suggests that the conflation of public health with migration policies can undermine public health goals and raise ethical issues. Participants in this study described facing a dilemma when they were unable to provide appropriate care to asylum seekers because of their legal status that entitles them only to care that ‘cannot be postponed’, a concept that is not clearly defined, leading to confusion and putting more responsibilities on health professionals. This law, which reflects an attempt to discourage the entry of new migrants, has been sharply criticized for violating the international human rights law (10, 34). Furthermore, despite awareness that migrants bear a disproportionate burden of infectious diseases in population terms, asylum seekers are housed far from screening units and in overcrowded conditions that increase the risk of transmission of infectious diseases among them (6). The outbreak of TB at one asylum seekers reception center is illustrative (35). In addition, the delay in screening described in this study adds to the problem as it can contribute to the spread of infection and make it difficult to ascertain whether some migrants were infected before migration or afterwards (9, 10). Finally promoting screening without adequate consideration of actual health and care needs, and guarantee of access to appropriate care and continuity of treatment does not only raise serious ethical issues, but is also counterproductive from a public health perspective (29). There is a need for further research to assess the effectiveness of the screening program and new approaches to offer screening in a more ethical way. A holistic and human rights based approach to migrant health that emphasizes vulnerability, as opposed to the threat of disease approach that has been traditionally used, is imperative for any effective public health policy promoting sustainable health outcomes (5, 18, 32).

### Study strengths and limitations

This study has strengths and limitations common to qualitative research. A purposive sample of informants from one profession, all interviewed by a single investigator, poses potential threats to credibility and transferability of findings. However, this study provides a rich and detailed description of the screening process from the perspectives of those who implement it, which strengthens the findings. Moreover, our participants were recruited in four different settings, a type of data source triangulation that enhanced checking information across informants. In addition, discussions among the research team enhanced the interview guide and style during the study and also data interpretation during the analysis process. The research design and its implementation as well as the author backgrounds have been reported to allow the reader to evaluate the relevance of data on which findings were based, the logic by which the conclusions were drawn, and the degree to which the interpretations reflect a coherent and grounded conclusion (16). These findings were also presented and discussed in seminars with other screening staff in other parts of Sweden who acknowledged it as persuasive. Finally, these findings only reflect the perspectives of health care providers, but this study is part of a larger research project that also includes studies with migrants and interpreters.

## Conclusions

These findings illustrate complex challenges that could limit access, uptake, and delivery of screening and thus compromise achievement of desired public health outcomes. They highlight the need for a multilevel strategy. This entails avoiding the conflation of migration with health policies and harmonizing existing policies and regulations to make health care services more accessible and acceptable to migrants, and facilitate health professionals’ efforts in promoting public health. It also highlights the need to improve the structure and coordination of the screening program through allocation of adequate resources and facilitation of collaboration among different agencies involved. However, the involvement of migration staff in the screening process is questionable and should be reconsidered. In addition, it emphasizes the need to improve the skills of all staff involved in understanding and effectively responding to the needs of migrants through continuing education. Finally, the need to improve migrants’ health literacy is emphasized. Outreach interventions using community health educators are warranted to educate migrants about health issues in appropriate languages with culturally relevant information and activities.
